# Clinical Characteristics and Risk Factors for Mortality in Cryptococcal Meningitis: Evidence From a Cohort Study

**DOI:** 10.3389/fneur.2022.779435

**Published:** 2022-04-29

**Authors:** Fengjuan Wang, Yu Wang, Jianqing He, Zhe Cheng, Shouquan Wu, Minggui Wang, Ting Niu

**Affiliations:** ^1^Department of Hematology and Research Laboratory of Hematology, West China Hospital, Sichuan University, Chengdu, China; ^2^Department of Respiratory and Critical Care Medicine, The First Affiliated Hospital of Zhengzhou University, Zhengzhou, China; ^3^Department of Respiratory and Critical Care Medicine, West China Hospital, Sichuan University, Chengdu, China

**Keywords:** cryptococcal meningitis, clinical characteristics, risk factors, India ink stain, cerebrospinal fluid culture

## Abstract

**Introduction:**

Despite advances in the diagnosis and management, cryptococcal meningitis (CM) is still associated with high mortality due to insufficient knowledge about clinical characteristics and risk factors for poor outcomes. The aim of the present study is to provide additional evidence for regarding clinical characteristics, diagnosis, and factors associated with increased risk of mortality in CM patients.

**Methods:**

In this cohort study, we included eligible patients consecutively admitted to West China Hospital between January 2009 and December 2018. The clinical characteristics and diagnosis method of cerebrospinal fluid culture and India ink stain were analyzed. Independent risk factors were identified by a multivariable logistic regression.

**Results:**

A total of 186 CM patients were included in the analysis. After a 1-year follow-up, 63 patients had died. Headache is the most common presenting symptom (97.3%), followed by vomiting (72%), fever (71.5%), altered consciousness (45.7%), abnormal vision (32.8%), and seizure (15.1%). Older age, altered consciousness or seizures, lower white blood cell count or total protein in cerebrospinal fluid (CSF), and unidentified CSF cryptococcal antigen (CrAg) are all factors associated with increasing risk of death (*P* < 0.05). We also found a dose-dependent trend between the number of symptoms and risk of death (trend *p* < 0.001). Multivariate logistic regression revealed that age (*P* = 0.004, OR = 1.042, 95% CI 1.013–1.071), seizure (*P* = 0.025, OR = 3.105, 95% CI 1.152–8.369), altered consciousness (*P* < 0.001, OR=6.858, 95% CI 3.063–15.38), and unidentified CSF CrAg are the independent prognostic factors. In addition, we observed that diagnosis of 28.5% and 22.5% CM could not be established by a single testing of CSF India ink stain or culture, respectively. Use of multiple testing methods or combination of the two assays increases the detection rate.

**Conclusion:**

Our data show that older age, seizures, altered consciousness, and an inability to detect CSF CrAg are the independent risk factors of death within 1 year in CM patients. Moreover, we recommend use of multiple testing methods with CSF culture and India ink stain. Combined testing with both assays should be considered for initial CM diagnosis.

## Introduction

Cryptococcal meningitis (CM), caused by *Cryptococcus* spp. infection, is a life-threatening opportunistic disease, which is annually responsible for over 220,000 cases and 181,000 deaths globally (1). The HIV pandemic has aggravated incidence of CM, leading to roughly 15% of HIV-related deaths (2). CM has also been reported in HIV-negative patients, which account for a major proportion in Asian populations (3, 4). Previous studies have reported 78% mortality in HIV-positive CM patients and 42% in HIV-negative patients (5).

A clear understanding of the clinical characteristics of CM is urgently required for early recognition and improvement of clinical outcomes. The most common symptoms of CM are fever along with signs associated with elevated intracranial pressure (e.g., headache, vomiting, and ocular involvement) (6). Moreover, some symptoms may be predictors of poor prognosis in HIV-positive CM patients (7). However, data is still lacking, especially for HIV-negative patients.

Cerebrospinal fluid (CSF) examination is necessary for CM diagnosis due to the nonspecific clinical features. Furthermore, there have been some sporadic reports of asymptomatic patients (8). CSF culture and India ink stain are still the main methods used for CM diagnosis in some resource-limited settings due to a lack of access to cryptococcal antigen (CrAg) lateral flow assay tests, which show high sensitivity (98.8%) and specificity (99.3%) (9, 10). This necessitates a detailed evaluation of CSF culture and India ink stain.

Herein, we describe the clinical features and analyze the death-related risk factors in CM and provide additional supporting evidence to aid clinical diagnosis and prognostic prediction.

## Method

### Study Design and Subjects

This cohort study included eligible patients consecutively admitted to West China Hospital between January 2009 and December 2018. All data were provided by the department of the information center of West China Hospital. We extracted the data including demographic characteristics, symptoms, complications, and the CSF testing of all the included patients. Subsequently, at least one 12-month follow-up was conducted for collecting the all-cause death. Ethical approval for this study was obtained from the Institutional Review Board of the West China Hospital of Sichuan University.

### Inclusion and Exclusion Criteria

**Figure 1:**
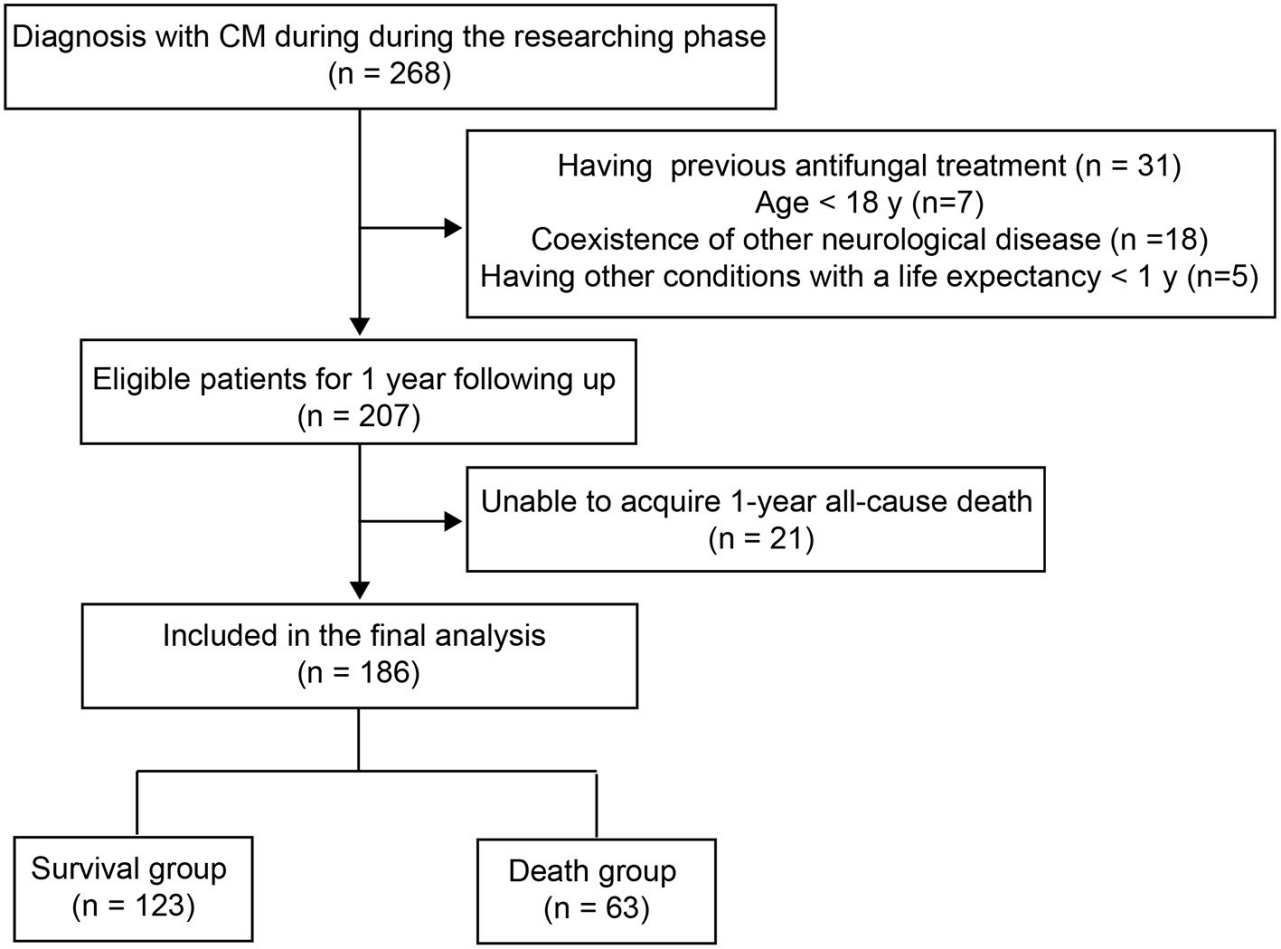
Flowchart of the study population.

The inclusion criteria were: (1) CM confirmation by CSF India ink stain and/or culture; (2) diagnosis with CM without antifungal treatment. Patients with the following criteria were excluded: (1) Age < 18 years; (2) other preexisting neurological diseases (e.g., neurological infection with tuberculosis, bacteria or virus, neurological cancer, and autoimmune-associated neurological disease); (3) other conditions with a life expectancy < 1 year; (4) incomplete clinical data or inability to acquire 1-year all-cause death. A schematic representation of this study is shown in Figure 1.

### Statistical Analyses

Statistical analyses were performed using R (version 3.6.1; Foundation for Statistical Computing). Continuous data were presented as mean ± standard deviation (SD) or as the median and interquartile range (IQR). Categorical variables were presented as the number of cases with frequency. Univariate difference between death group and survival group was analyzed using independent *T*-test, Mann-Whitney U test, Chi-square test or Fisher's exact test, according to the characteristic of the variable. Multivariable logistic regression was conducted including possible prognostic factors (*P* < 0.05 in univariate analysis) and HIV co-infection, to provide visualized risk prediction using R with “rms” and “UpSerR” packages. The final figure assembly was performed in Adobe Illustrator (Adobe, San Jose, CA). Statistical significance was established with a two-sid *P* < 0.05.

## Results

### Demographic and Clinical Characteristics

Of the 268 CM patients screened for this study, 186 cases were included in the final analyses (Figure 1). Following the 1-year follow-up (median, [Q1, Q3]: 15 months, [13.5-18 months]), 63 subjects (33.9%) were deceased. The demographic characteristics of the survival and death groups are presented in Table 1. The mean age at diagnosis was 43.57 (16.25) years for individuals who were alive after 1 year versus 49.5 (15.6) years for those who had died before the one-year follow-up (*p* = 0.018).

**Table 1:** Baseline characteristics of the subjects.

**Characteristics**	**All subjects, *n* = 186**	**Survival group, *n* = 123**	**Death group, *n* = 63**	***P***
Age, mean ± SD	45.58 ± 16.24	43.57 ± 16.25	49.51 ± 15.60	0.018^†^
Male, *n* (%)	107 (57.5)	66 (53.7)	41 (65.1)	0.136^#^
**Ethnicity**
Chinese Han population, *n* (%)	179 (96.2)	120 (97.6)	59 (93.7)	0.230^*^
Tibetan, *n* (%)	5 (2.7)	2 (1.6)	3 (4.7)	
Yi, *n* (%)	2 (1.1)	1 (0.8)	1 (1.6)	
**Complication**
HIV infection, *n* (%)	57 (30.6)	33 (26.8)	24 (38.1)	0.115^#^
DM, *n* (%)	18 (9.7)	12 (9.8)	6 (9.5)	0.960^#^
Hepatitis, *n* (%)	20 (10.8)	14 (11.4)	6 (9.5)	0.699^#^
Rheumatism, *n* (%)	15 (8.1)	12 (9.8)	3 (4.8)	0.236^#^
CRF, *n* (%)	11 (5.9)	8 (6.5)	3 (4.8)	0.752^#^

*P-value was calculated by comparing the survival group with death group*.

† *Calculation using T-test*.

# *Calculation using Chi-square test*.

* *Calculation using Fisher's exact test (Han vs. non-Han Chinese). HIV, human immunodeficiency virus; DM, diabetes mellitus; CRF, chronic renal failure*.

Males (57.5%) and Chinese Han (96.2%) were predominant among these subjects. However, no statistical differences were observed related to survival based on gender or race (*P* = 0.136 and 0.230, respectively). The most common comorbidity in CM was HIV co-infection (30.6%), followed by hepatitis (10.8%), and diabetes (9.7%). We found no statistical differences with comorbidity and survival, although we observed more HIV infection cases in the death group (38.1 vs. 26.8%).

**Figure 2:**
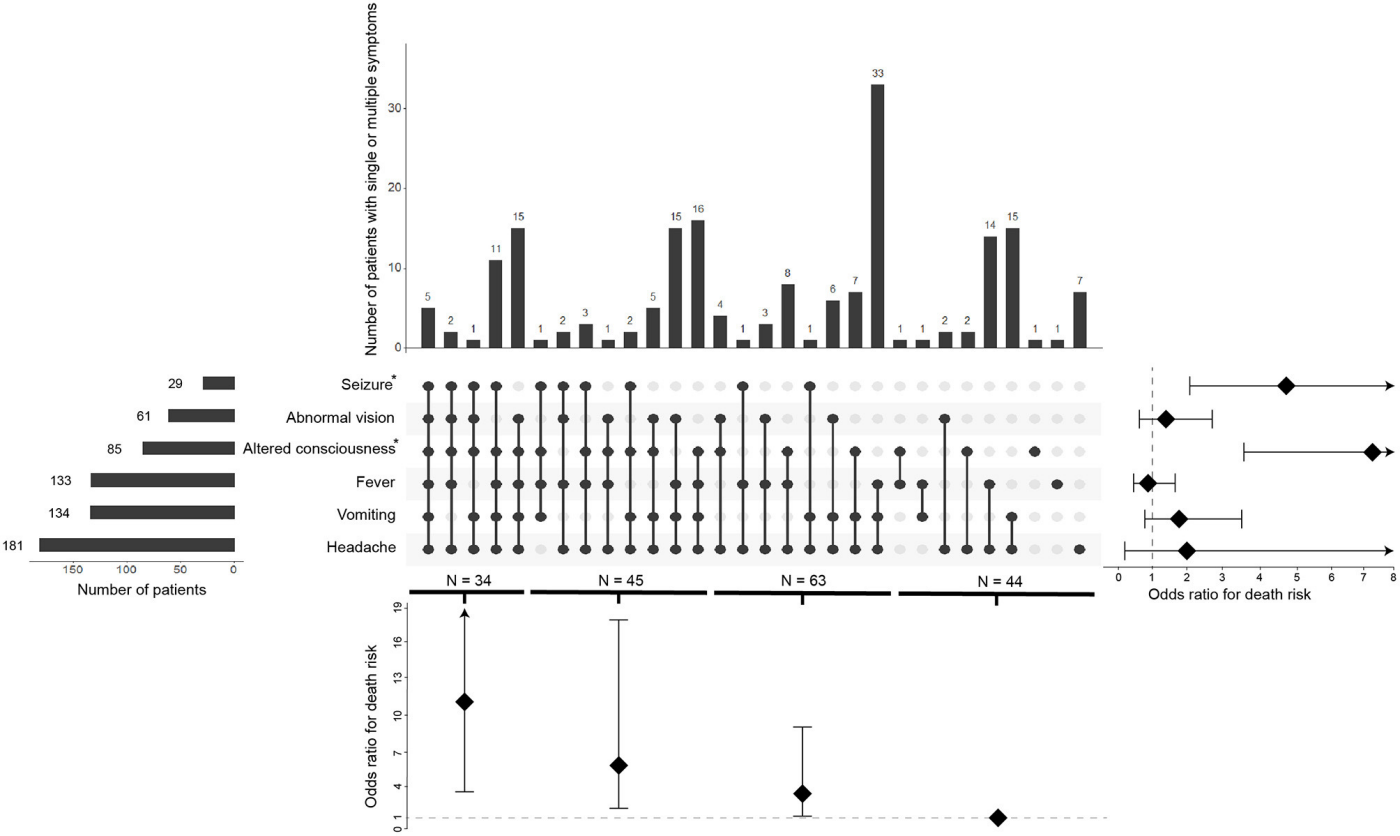
Overlap of the six common symptoms and its correlation of death risk. The visualized intersection of six symptoms (seizure, abnormal vision, altered consciousness, fever, vomiting, headache) sets as a matrix in which the rows represent the different symptoms, and the columns represent their intersections. For each symptom that is part of a given intersection, a black-colored dot is placed in the corresponding matrix cell. If a symptom is not part of the intersection, a light, gray-colored dot is shown. A vertical black line connects the topmost black dot with the bottommost black dot in each column to emphasize the overlapping relationships. The bar chart above of the matrix displays the size of each overlapping. The bar chart to the left of the matrix displays the size of each symptom. The forest plot of odds ratio for death risk regarding each symptom is shown to the right of the matrix. The forest plot of odds ratio for death risk regarding the overlapping is shown below the matrix.

The six most common clinical symptoms at the time of CM diagnosis are shown in Figure 2. Headache is the most common presenting symptom (97.3%), followed by vomiting (72%), fever (71.5%), altered consciousness (45.7%), abnormal vision (32.8%), and seizure (15.1%). We observed that altered consciousness or seizure, which occur in 45.7 or 15.6% of cases, respectively, is associated with an increased risk of death (*P* < 0.001, OR = 7.438, 95% CI 3.708–14.92; *P* < 0.001, OR = 4.880, 95% CI 2.104–11.32, respectively). Moreover, considering the 44 patients with one and two symptoms as a reference, the death associated OR was 3.12 (95% CI 1.06–9.18) for the patients with three symptoms, 6.24 (95% CI 2.08–18.77) for four symptoms, and 11.14 (95% CI 3.51–35.36) for five and six symptoms. We found a dose-dependent effect between the number of symptoms and the risk of death (trend *p* < 0.001) (Figure 2).

### CSF Testing

The CSF opening pressure, cytology, and biochemical parameters are listed in Table 2. We found that the proportion of cases with opening pressure ≥180 mm H_2_O is higher in the group of patients that died (82 vs. 73.7%). However, the difference was not statistically significant (*P* = 0.218). Further, we observed a lower white blood cell (WBC) count and total protein in CSF of patients that died compared to those that survived (*P* < 0.001 and *P* = 0.002, respectively). In contrast, differences in the chloride and glucose content in the CSF between both groups showed no statistically significant differences. We also found that the proportion of the determination of CSF CrAg was much higher in patients who survived versus those that died (33.3 vs. 9.5%, *p* < 0.001).

**Table 2:** Comparison of CSF features in survival group and death group.

**Variables**	**Survival group (*n* = 123)**	**Death group (*n* = 63)**	***P***
Opening pressure ≥180 mm H_2_O, *n* (%)	87 (73.7)	50 (82.0)	0.218^†^
WBC count /μl, median (IQR)	65 (200)	10 (30)	<0.001^#^
Total protein g/dl, median (IQR)	0.87 (0.83)	0.61 (0.51)	0.002^#^
Chloride mmol/L, median (IQR)	118 (9.3)	119 (7.7)	0.287^#^
Glucose mmol/L, median (IQR)	1.46 (1.78)	1.99 (1.85)	0.925^#^
CrAg detection, *n* (%)	41 (33.3)	6 (9.5)	<0.001^†^

† *Calculation using Chi-square test*.

# *Calculation using Mann-Whitney U test. IQR, interquartile range; CSF, cerebrospinal fluid; WBC, white blood cell; CrAg, cryptococcal antigen*.

**Figure 3:**
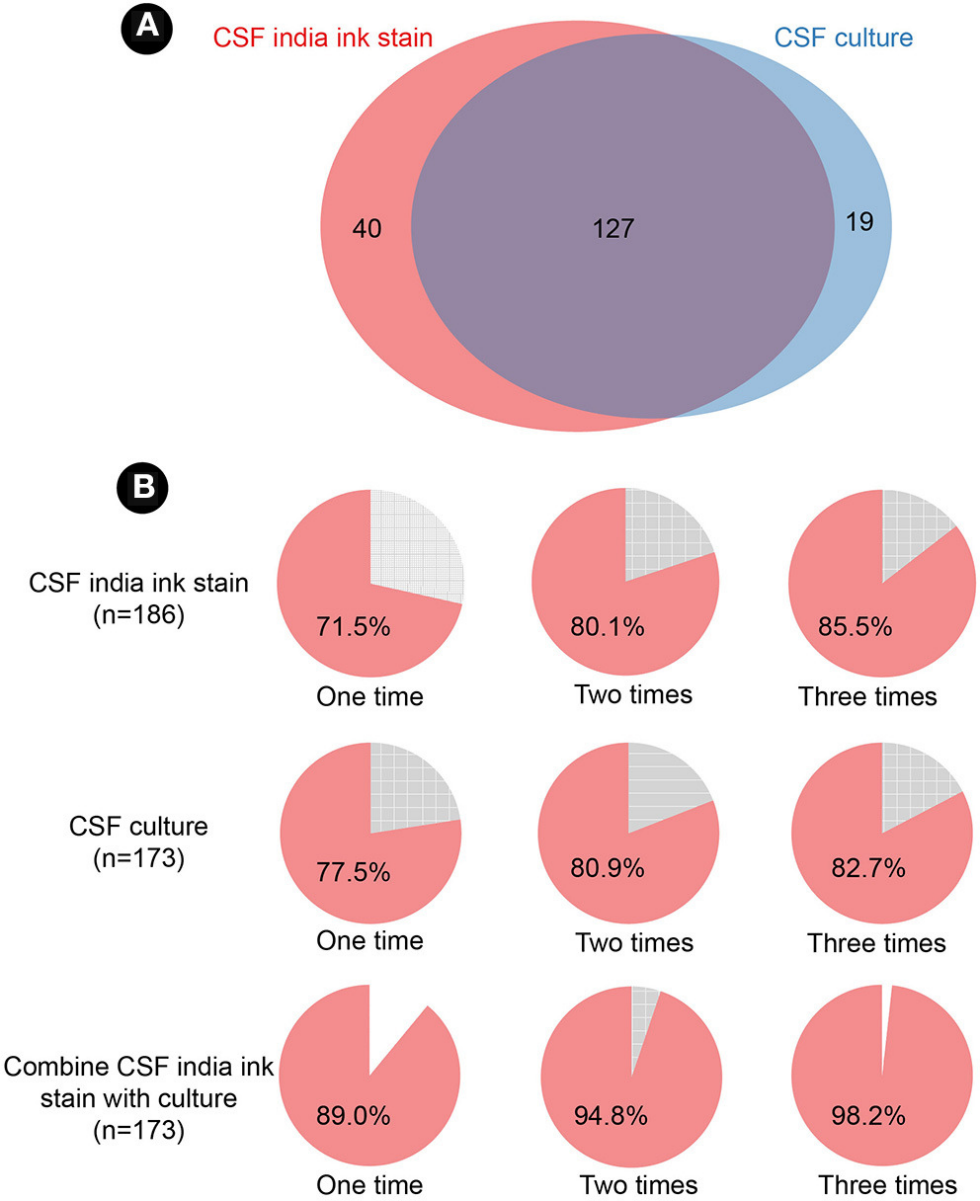
Cerebrospinal fluid India ink stain and culture. **(A)** Venn diagram of India ink stain and culture. **(B)** positive detection of India ink stain, culture, and combination of the two assays. CSF, cerebrospinal fluid.

Among the 186 patients, we found 40 cases with only positive India ink stain, 19 patients with only positive CSF culture, and 127 patients that were positive in both assays (Figure 3A). The time to positive diagnosis of CM by culture and India ink stain was 6.3 ± 4.4 days and 3.7 ± 5.3 days, respectively. Furthermore, diagnosis of 28.5 and 22.5% of CM cases could not be established by a single test with CSF India ink stain or culture. If multiple testing is performed, diagnosis can be established in ~80% of cases with two tests, or in 85.5 and 82.7% of cases with three times tests of India ink stain and culture, respectively (Figure 3B). Additionally, 89% of the cases can be diagnosed by combining the CSF India ink stain and culture. Moreover, identification of positive cases can be increased to 94.8 and 98.2% using double and triple testing, respectively, with this dual CSF India ink stain/culture diagnostic method (Figure 3B).

### Multiple Logistic Regression

For multiple logistic regression, we chose the candidate risk variables with *P* < 0.05 in univariate difference analyses, with gender, race and comorbidities set as covariates. We identified age (*P* = 0.004, OR = 1.042, 95% CI 1.013–1.071), seizure (*P* = 0.025, OR = 3.105, 95% CI 1.152–8.369), altered consciousness (*P* < 0.001, OR = 6.858, 95% CI 3.063–15.38), and inability to identify CSF CrAg (*P* = 0.001, OR = 0.209, 95%CI 0.081–0.541) as independent risk factors of death in CM patients (Table 3).

**Table 3:** Multivariable logistic regression of candidate variables for death risk in patients with cryptococcal meningitis.

**Variables**	**OR**	**95%CI**	***P***
Age	1.042	1.013–1.071	0.004
Seizure	3.105	1.152–8.369	0.025
Altered consciousness	6.858	3.063–15.38	<0.001
CSF WBC count	1	1.000–1.001	0.188
CSF total protein	0.665	0.359–1.230	0.193
CSF cryptococcal antigen testing	0.209	0.081–0.541	0.001

*P was calculated suing multivariable logistic regression, which adjusted by gender, race, complication with human immunodeficiency virus infection, diabetes mellitus, hepatitis, rheumatism, chronic renal failure. CSF, Cerebrospinal fluid; WBC, white blood count*.

## Discussion

This is a comprehensive clinical study examining factors that affect clinical outcomes in CM patients and the detection in the CSF. Present results emphasize a high mortality rate in patients with CM. Early recognition of the risk factors may allow early interventions to potentially mitigate poor outcomes. However, previous studies have mainly involved subjects with HIV and CM coinfection in a high HIV burden setting due to increased susceptibility to CM in HIV patients (11). In addition, previous work has also shown that population heterogeneity affects the immune response to the pathogen, resulting in altered clinical symptoms and prognoses (12, 13). Recently, a novel predictive model was established for calculating one-year mortality in CM (14). However, the evidence is still inadequate due to the small sample size.

The present study included the CM patients up to 10 years with a follow-up period of at least 1 year in west China. A total of 186 pathogen confirmed CM patients were finally analyzed. We found that headache is the most common symptom, followed by vomiting and fever, which is similar to previous studies (15, 16). Previous studies have also reported that conditions that may be associated with impaired immune function are frequently seen in CM patients (17).

Despite requiring a long detection time, CSF culture is considered the gold standard in CM diagnosis. India ink stain is a rapid and economical diagnostic method for CM but it is limited by low sensitivity (18). To our acknowledge, there is no available data to show the number of times that either of these tests needs to be replicated. Herein, we show that either culture or India ink stain need to be repeated at least twice to reach an 80% positive rate. A superior approach is to combine these two diagnostic methods. We found that seizure and altered consciousness are associated with an increased risk of 1-year death in CM, which is consistent with previous findings (19, 20). Moreover, we observed that the number of symptoms shows positive correlation with the risk of death. Therefore, we recommend that symptoms, especially seizure and altered consciousness, should be a main focused in the course of the illness. CSF Examination of the CSF is essential in evaluating the disease state during host-pathogen interactions in *Cryptococcus* spp. infection (21). Blood-brain barrier disruption and inflammatory infiltration caused by invading pathogens may result in a dramatic change in the CSF (22). We also found that lower WBC count and total protein were associated with an increased risk of death in univariate analysis, which is consistent with previous studies (23). A lower WBC count and total protein of CSF may be indicative of an insufficient inflammatory response, leading to immune evasion and, subsequently, increased replication and dissemination of *Cryptococcus* spp. (22). Interestingly, some studies have published conflicting data (24). A possible explanation may account for the stage of disease progression because parameters in the CSF may change as disease progresses and therapeutic interventions may also affect this. In our study, to avoid confounding factors caused by disease stage and drugs, the subjects were first diagnosed without antifungal therapy. However, some previous studies did not include such a discriminating design. Screening of CrAg is recommended for early diagnosis with CM due to its high sensitivity and availability (9, 10). However, its widespread application is limited by cost and the logistical issues. In the present study, we found that mortality is significant lower in patients who had been diagnosed using the CSF CrAg method. However, this finding should be viewed with caution, because the majority of patients who were screened using the CSF CrAg method were in the last few years of the study period (after 2013). The potential confounders brought by the enhanced acknowledgment and improvement of therapeutic intervention of CM in recent years is unavoidable. In the multivariable logistic regression, we found the age, seizure, altered consciousness, and inability to identify CSF CrAg are independent risk factors of increased death in CM patients. Statistical differences in WBC count or the total CSF protein were removed. This might be due to the collinearity in these variables. Therefore, older patients and manifestation of seizures and altered consciousness should be paid close attention in clinical practice.

We recognize that our study also had several weaknesses. Firstly, 70–90% of CM cases occur in HIV-negative patients (25). In the present study, HIV-positive subjects merely account for 30.6% of cases, which might affect the generalization of these data. However, reports of HIV-negative CM patients are increasing (26). Recent retrospective population-based studies found the incidence of Cryptococcosis among HIV-negative patients to be close to half of the overall reported cases (between 44 and 55%) (27, 28). In addition, to better explain the relationship between CM and death, and limit the affect of the CSF by other conditions, we excluded the coexistence of other neurological disease, which may lead to a lower mortality than previous studies. This is not an interventional experiment. Thus, the causal relationship of poor outcome with the risk factors cannot be unequivocally demonstrated. Nevertheless, we tried our best to reduce confounding factors and allow easier comparison between the both groups. Additionally, the 10 year accumulation of sample size offered sufficient statistical power. Ultimately, our study demonstrated stably that age, seizure, and altered consciousness are associated with increased risk of 1-year death in CM patients using multiple logistic regression. Another limitation is our study was a single-center study. Besides highlighting the risk factors for death in CM patients, we firstly provided data to support the necessity for multiple testing in CSF culture and India ink stain and combining these two assays. However, the results should be replicated in a large multicenter trial.

In conclusion, the results highlight that age, seizure, and altered consciousness are associated with increased 1-year death risk in CM patients. Further, we recommend multiple testing in CSF culture and India ink stain, and the combination of these two assays should be considered for initial diagnosis of CM.

## Data Availability Statement

The original contributions presented in the study are included in the article/supplementary material, further inquiries can be directed to the corresponding author.

## Ethics Statement

The studies involving human participants were reviewed and approved by Institutional Review Board of the West China Hospital of Sichuan University. The patients/participants provided their written informed consent to participate in this study.

## Author Contributions

FW and YW had full access to all the data in the study and take responsibility for the integrity and accuracy of the data analysis. TN contributed to study concept and design. The first draft of the manuscript was written by FW. JH and ZC performed the critical revision of the manuscript for important intellectual content. SW and MW performed the data collection. All authors contributed to the article and approved the submitted version.

## Funding

This work was supported by the grants from National Natural Science Foundation of China (No. 82000015).

## Conflict of Interest

The authors declare that the research was conducted in the absence of any commercial or financial relationships that could be construed as a potential conflict of interest.

## Publisher's Note

All claims expressed in this article are solely those of the authors and do not necessarily represent those of their affiliated organizations, or those of the publisher, the editors and the reviewers. Any product that may be evaluated in this article, or claim that may be made by its manufacturer, is not guaranteed or endorsed by the publisher.
